# Abiotic and biotic factors responsible for antimonite oxidation in *Agrobacterium tumefaciens* GW4

**DOI:** 10.1038/srep43225

**Published:** 2017-03-02

**Authors:** Jingxin Li, Birong Yang, Manman Shi, Kai Yuan, Wei Guo, Qian Wang, Gejiao Wang

**Affiliations:** 1 State Key Laboratory of Agricultural Microbiology, College of Life Science and Technology, Huazhong Agricultural University, Wuhan 430070, P.R. China

## Abstract

Antimonite [Sb(III)]-oxidizing bacteria can transform the toxic Sb(III) into the less toxic antimonate [Sb(V)]. Recently, the cytoplasmic Sb(III)-oxidase AnoA and the periplasmic arsenite [As(III)] oxidase AioAB were shown to responsible for bacterial Sb(III) oxidation, however, disruption of each gene only partially decreased Sb(III) oxidation efficiency. This study showed that in *Agrobacterium tumefaciens* GW4, Sb(III) induced cellular H_2_O_2_ content and H_2_O_2_ degradation gene *katA*. Gene knock-out/complementation of *katA, anoA, aioA* and *anoA/aioA* and Sb(III) oxidation and growth experiments showed that *katA, anoA* and *aioA* were essential for Sb(III) oxidation and resistance and *katA* was also essential for H_2_O_2_ resistance. Furthermore, linear correlations were observed between cellular H_2_O_2_ and Sb(V) content *in vivo* and chemical H_2_O_2_ and Sb(V) content *in vitro* (R^2^ = 0.93 and 0.94, respectively). These results indicate that besides the biotic factors, the cellular H_2_O_2_ induced by Sb(III) also catalyzes bacterial Sb(III) oxidation as an abiotic oxidant. The data reveal a novel mechanism that bacterial Sb(III) oxidation is associated with abiotic (cellular H_2_O_2_) and biotic (AnoA and AioAB) factors and Sb(III) oxidation process consumes cellular H_2_O_2_ which contributes to microbial detoxification of both Sb(III) and cellular H_2_O_2_.

Antimony (Sb) is an element belonging to Group 15 of the Periodic Table and behaves similar to arsenic (As). Sb and its compounds are recognized as priority pollutants by the United States Environmental Protection Agency^1^ and European Union^2^. In recent years, the serious Sb pollution resulting from increased exploitation and industrial emission has aroused growing concern^3^,^4^,^5^. Among various oxidation states (−3, 0, 3, and 5), antimonite [Sb(III)] and antimonate [Sb(V)] are the most common forms^6^. Because microbial redox reactions can be used as a strategy for biochemical detoxification and can further affect the mobility, toxicity, and bioavailability of Sb in the environment^7^,^8^, a better comprehension of the microbe-Sb interactions is important for the bioremediation of Sb-contaminated environments and to understand the Sb biogeochemical cycle. In generally, Sb(III) is more toxic than Sb(V)^5^,^9^, thus, examining the mechanisms driving bacterial oxidation from Sb(III) to Sb(V) could be of significant value in this regard.

The understanding of microbial Sb transformation remains deficient compared to that of As^10^. It has been reported that the glycerol transporter GlpF and its homolog Fps1p are responsible for Sb(III) uptake, reflecting the structural similarities between Sb(OH)_3_ and glycerol^11^,^12^,^13^, while the As(III) efflux proteins ArsB and Acr3 can also function as Sb(III) efflux pumps^14^,^15^. Nevertheless, the pathway of Sb(V) entrance has not been found yet. In addition, the genes and enzymes involved in microbial Sb(V) reduction and Sb(III) methylation have not been identified, although these phenomena are environmentally widespread^9^.

In contrast with bacterial As(III) oxidation, which has been clarified for several decades, the mechanism of bacterial Sb(III) oxidation is of relatively recent interest. At present, about 60 Sb(III)-oxidizing bacteria, widely existing in various genera (e.g., *Pseudomonas, Comamonas, Agrobacterium* and *Acinetobacter*), have been reported^16^. Recently, we found that the As(III) oxidase AioAB is also function as an Sb(III) oxidase in *Agrobacterium tumefaciens* 5A^17^, and subsequently we found a novel Sb(III) oxidase AnoA belonging to the short-chain dehydrogenase/reductase family of enzymes in *A. tumefaciens* GW4^18^. Compared with *A. tumefaciens* 5A^17^, strain GW4 has considerably higher Sb(III) resistance and Sb(III) oxidation efficiency^18^. However, deletion of each gene only partially influenced the Sb(III) oxidation efficiency of *A. tumefaciens* strains, indicating other unknown mechanisms.

Chemically, Sb(III) can be oxidized through several oxidants, such as H_2_O_2_, iodate, nature minerals (e.g., Fe and Mn oxyhydroxides) and humic acid under oxic conditions^19^,^20^,^21^,^22^. In bacterial cells, the aberrant electron flow under stress conditions from the electron transport chain or cellular redox enzymes to O_2_ results in the production of reactive oxygen species (ROS)^23^. The harmful ROS [e.g., superoxide (O_2_^**·**−^), hydroxyl (OH^**·**^) and H_2_O_2_] can induce DNA damage and the oxidative deterioration of lipids and proteins^24^,^25^,^26^. Thus, bacteria have evolved defense mechanisms against the oxidative stress. Superoxide dismutase (Sod), which catalyzes the dismutation of O_2_^**·**−^ to H_2_O_2_ and O_2_, plays an important role in defense against ROS. The generated H_2_O_2_ is subsequently consumed by catalases and peroxidases^23^,^27^. In a recent work, we deleted the catalase gene *katA* in *A. tumefaciens* GW4 and observed that the Sb(III) oxidation efficiency of the mutant strain was significantly increased, and the phenotype of the complementary strain was recovered^16^. Moreover, the transcription of *katA* was induced by both H_2_O_2_ and Sb(III)^16^. Therefore, we proposed that the increased Sb(III) oxidation efficiency in the mutant strain might reflect the accumulation of H_2_O_2_ in bacterial cells. Nevertheless, there is no direct evidence of a correlation between H_2_O_2_ and Sb(III) oxidation.

In the present study, we performed gene knock-out/complementation of *katA, anoA, aioA* and *anoA/aioA* and in combination with the analyses of Sb(III) oxidation, cellular H_2_O_2_ content and resistance of Sb(III) and H_2_O_2_ in *A. tumefaciens* GW4^28^. We provide the first evidence that besides the biotic factors (AnoA and AioAB), the Sb(III) induced cellular H_2_O_2_ also catalyzes bacterial Sb(III) oxidation as an abiotic oxidant. The present study documented the abiotic Sb(III) oxidation and clarified the relationship between Sb(III) resistance and bacterial oxidative stress. The results represent an important step toward unraveling the co-metabolism of bacterial Sb(III) oxidation.

## Results

### Genomic information of *aioA, anoA* and *katA* in *A. tumefaciens* GW4

**Figure 1:**
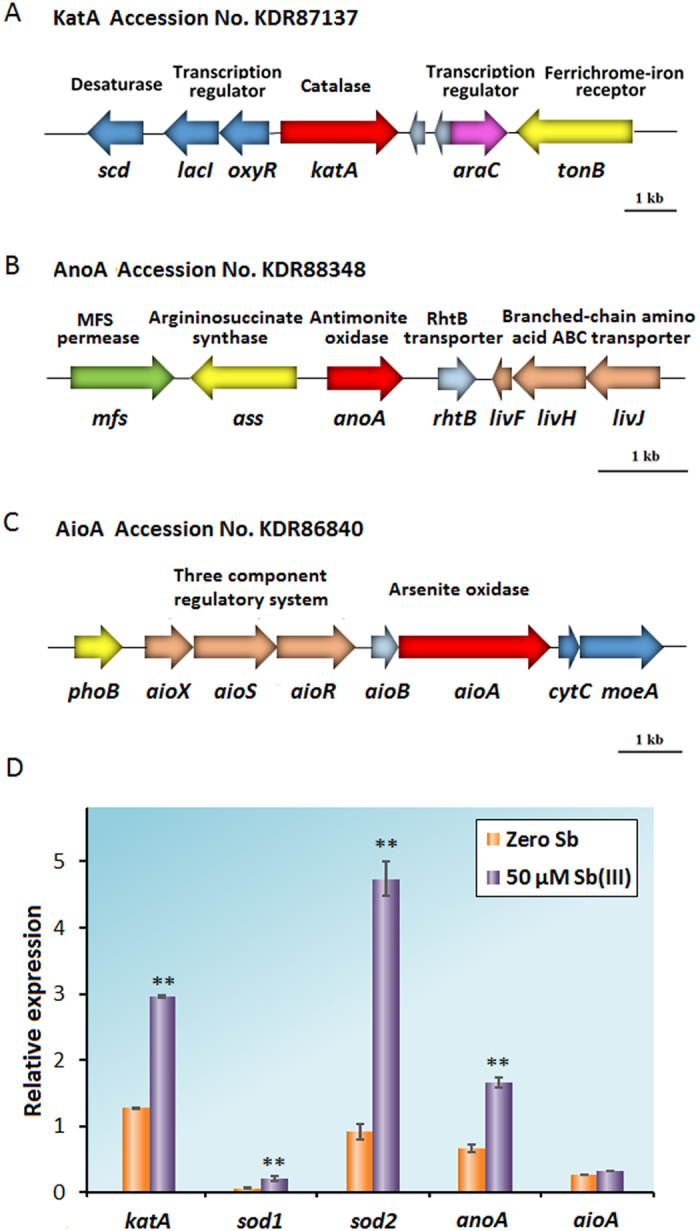
Physical map of *katA, anoA* and *aioA* (**A–C**) and gene transcription of *katA, sod1, sod2, anoA* and *aioA* in *A. tumefaciens* GW4 (**D**). (**A–C**) Gene clusters of *katA, anoA* and *aioA.* (**D**) Quantitative reverse transcriptase-PCR analysis. Total RNA was isolated from strain GW4 cultured in CDM medium with 0.5 h induction of 0 or 50 μM Sb(III). The *16S rRNA* gene was used as a reference. Data are shown as the mean of three replicates, with the error bars representing ± SD. **Represents p < 0.01; *represents p < 0.05.

*A. tumefaciens* GW4 is an Sb(III)-oxidizing strain, and its genome sequence was previously published (Accession No. AWGV00000000)^18^. To investigate the abiotic factors of Sb(III) oxidation, two types of cellular oxidative stress-related genes were analyzed. The catalase gene *katA*, responsible to degrade H_2_O_2_ to H_2_O and O_2_^27^, showed a 91% sequence identity with *katA* in *A. tumefaciens* C58^29^. We also analyzed the superoxide dismutase Sod, which could convert O_2_^**·**−^ to H_2_O_2_ and O_2_. The BlastN results showed that *katA* is a single-copy gene in the genome of strain GW4, while *sod* has two copies (*sod1* and *sod2*). Thus, subsequent gene knock-out and complementation studies associated with the abiotic factors of Sb(III) oxidation were mainly focused on the *katA* gene. The arrangement of *katA* and its surrounding genes are shown in Fig. 1A. For biotic Sb(III) oxidation, the oxidoreductase gene *anoA* (Fig. 1B), identified as a novel Sb(III) oxidase, is conserved in the genomes of *Agrobacterium, Sinorhizobium* and *Rhizobium* strains^18^. Although several genes were annotated as “short chain dehydrogenase”, “oxidoreductase”, or “putative oxidoreductase” in the genome of strain GW4, the BlastN analyses indicated that the sequences of these genes showed no similarities with that of *anoA*. Moreover, BlastP analyses showed that the protein sequence of AnoA showed only ~30% similarity with those of other oxidoreductases. In addition, draft genome sequencing revealed that an arsenic gene island located in contig 215 contains the As(III) oxidase genes *aioAB* (Fig. 1C), and *aioA* encodes the large subunit of As(III) oxidase^30^.

### Sb(III) induces the transcription of *katA, sod1, sod2* and *anoA*, but not *aioA*

To investigate the biotic and abiotic factors associated with Sb(III) oxidation in *A. tumefaciens* GW4, the transcription levels of genes *katA, sod1, sod2, anoA* and *aioA* were examined. The catalase KatA and superoxide dismutase Sod are involved in the bacterial oxidative stress response, and the Sb(III) oxidase AnoA and As(III) oxidase AioAB were both reported to catalyze Sb(III) oxidation *in vitro*^16^,^17^,^18^. The quantitative reverse transcriptase PCR assays indicated that the transcription levels of both *katA* and *anoA* were increased with the addition of Sb(III), consistent with the results of our previous studies^16^,^17^,^18^. In addition, the transcription of *sod1* and *sod2* were also induced by Sb(III). The transcription level of *sod1* was much lower than *sod2*, suggesting that *sod2* might play a more important role in dismutation of O_2_^**·**−^. However, the transcription level of *aioA* was not induced by Sb(III) (Fig. 1D), consistent with the previous observations in *A. tumefaciens* 5A^17^.

### Gene knock-out and complementation analyses showed effects of *katA, anoA* and *aioA* on Sb(III) oxidation

**Figure 2:**
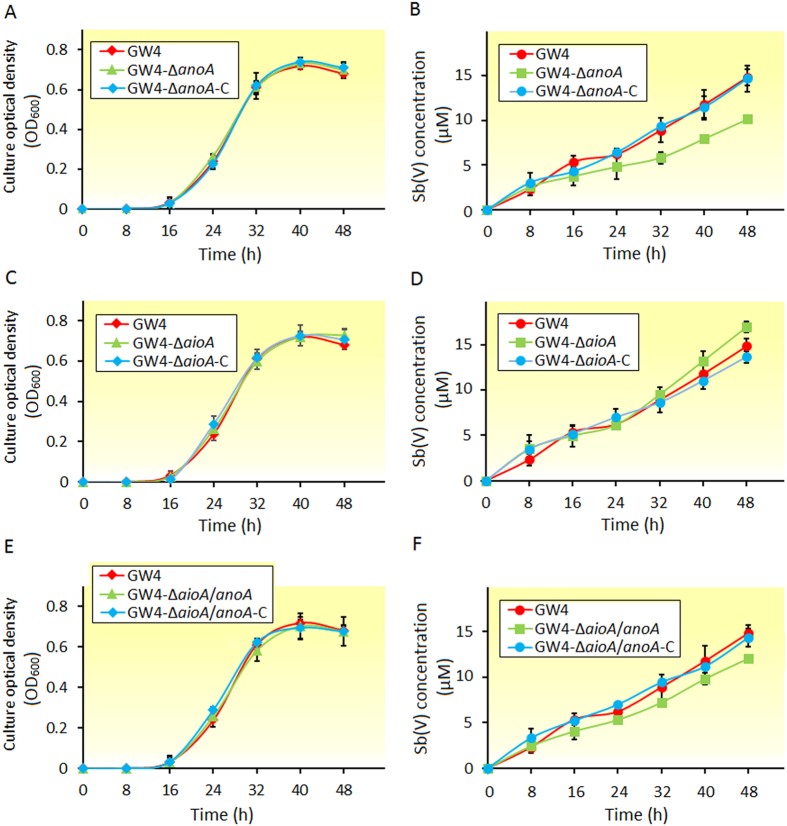
Growth and Sb(III) oxidation curves of *A. tumefaciens* strains. (**A**,**C**,**E**) The growth curves of *A. tumefaciens* strains in CDM medium containing 50 μM Sb(III). Panels A, C, E share the same data of strain GW4. (**B**,**D**,**F**) Sb(III) oxidation profiles of the same strains. Panels B, D, F share the same data of strain GW4. The culture conditions were the same as previous described (Li *et al*.^15^). Cell growth was measured based on culture optical density, and Sb(V) concentrations in the culture fluids were measured using HPLC-HG-AFS. Error bars correspond to the standard deviations of the means from three independent experiments. The Sb(III) oxidation efficiency was calculated at 48 h according to the formula: [Sb(V) concentration/Total Sb concentration]*100%.

Previously, we showed that the disruption of H_2_O_2_ degradation gene *katA* increased Sb(III) oxidation efficiency in strain GW4^16^. The successful deletion and complementation of *katA* were confirmed by diagnostic PCR shown in Fig. S1. Strains GW4-Δ*aioA*, GW4-Δ*anoA* and their complemented strains were obtained from previous studies^17^,^18^. Strains GW4-Δ*aioA*/*anoA* and GW4-Δ*aioA*/*anoA-*C were obtained from this study and diagnostic PCR and DNA sequencing were used to confirm the successful deletion and complementation (data not shown). Based on our previous studies^16^ and the growth tests in this study, all of the strains showed consistent growth profiles in CDM medium containing 50 μM Sb(III) (Fig. 2), indicating that the Sb(III) oxidation was not affected by the growth of the strains under 50 μM Sb(III). Based on our previous results^16^, we calculated that the Sb(III) oxidation efficiency of strain GW4-Δ*katA* (~52%) was increased by ~80% compared with the wild-type strain GW4 (~29%). The catalase KatA is responsible for cellular H_2_O_2_ consumption^27^, thus we proposed that the high efficient Sb(III) oxidation in strain GW4-Δ*katA* might be associated with the cellular H_2_O_2_ content. Moreover, the GW4-Δ*anoA* showed a ~30% decrease in the Sb(III) oxidation efficiency (Fig. 2B), which is similar to our previous study^18^. In contrast, deletion of *aioA* had no effect on the Sb(III) oxidation efficiency during the log phase, while the Sb(III) oxidation efficiency was slightly increased during the stationary phase (Fig. 2D). It has been suggested that in the stationary phase of bacterial growth, other Sb(III) oxidation mechanism(s) might exist and function more efficiently in the absence of *aioA*. The simultaneous deletion of *aioA* and *anoA* resulted in a phenotype of Sb(III) oxidation efficiency between GW4-Δ*aioA* and GW4-Δ*anoA* (~19% decreased) (Fig. 2F), and all of the complemented strains showed a Sb(III) oxidation efficiency similar to that of the wild-type strain GW4 (Fig. 2).

### The *katA, anoA* and *aioA* influence each other and further affect Sb(III) oxidation

**Figure 3:**
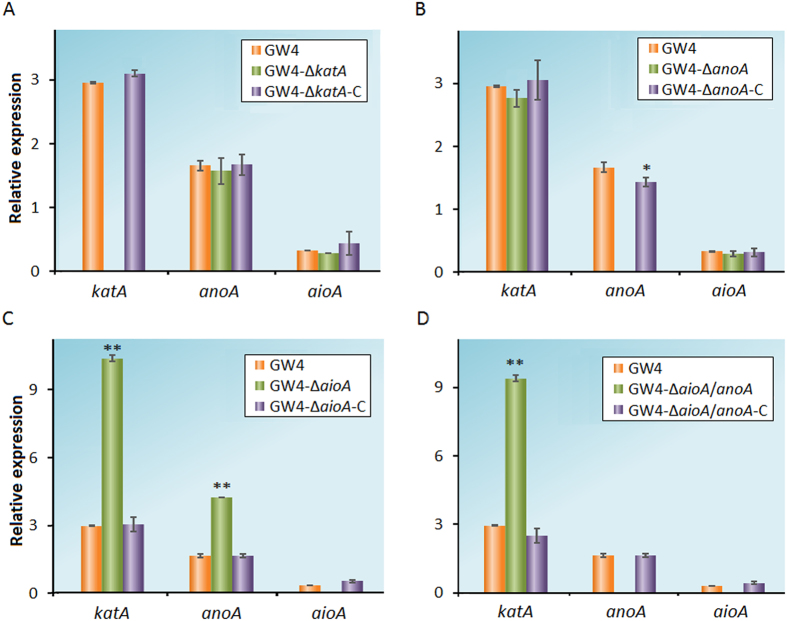
Quantitative reverse transcriptase-PCR analysis of the genes associated with Sb(III) oxidation in *A. tumefaciens* strains. (**A–D**) Total RNA was each isolated from strains GW4, GW4-Δ*katA,* GW4-Δ*katA-*C, GW4-Δ*anoA*, GW4-Δ*anoA*-C, GW4-Δ*aioA*, GW4-Δ*aioA-*C, GW4-Δ*aioA/anoA* and GW4-Δ*aioA/anoA-*C cultured with 50 μM Sb(III). Panels A-D share the same data of strain GW4. The 16S rRNA gene was used as a reference. Data are shown as the mean of three replicates, with the error bars representing ± SD. **Represents p < 0.01; *represents p < 0.05.

To elucidate how *katA, anoA* and *aioA* influence each other and further affect Sb(III) oxidation, we detected the transcription level of these genes in each *A. tumefaciens* strain. Bacterial cells were cultivated in CDM medium, and samples were collected after 0.5 h of induction with 50 μM Sb(III). In strain GW4-Δ*katA*, the transcription levels of *aioA* and *anoA* were not increased (Fig. 3A), suggesting that the reduced consumption of H_2_O_2_ might be responsible for the efficient Sb(III) oxidation. In addition, the transcription levels of *aioA* and *katA* in strain GW4-Δ*anoA* showed no significant difference with the wild-type strain, consistent with the phenotype of decreased Sb(III) oxidation efficiency (Fig. 3B). In contrast, the transcription levels of *anoA* and *katA* were up-regulated in strain GW4-Δ*aioA* (p < 0.01), indicating that the AnoA- and H_2_O_2_-catalyzed Sb(III) oxidation was enhanced relative than the wild-type strain (Fig. 3C). Therefore, the deletion of *aioA* increased the Sb(III) oxidation efficiency in strain GW4. In strain GW4-Δ*aioA*/*anoA*, although the transcription level of the *katA* was increased (p < 0.01), the loss function of AnoA could not be compensated (Fig. 3D). Expectedly, all of the complemented strains recovered the phenotype back to the wild-type strain GW4 (Fig. 3).

### The *katA, anoA* and *aioA* are involved in Sb(III) resistance

**Figure 4:**
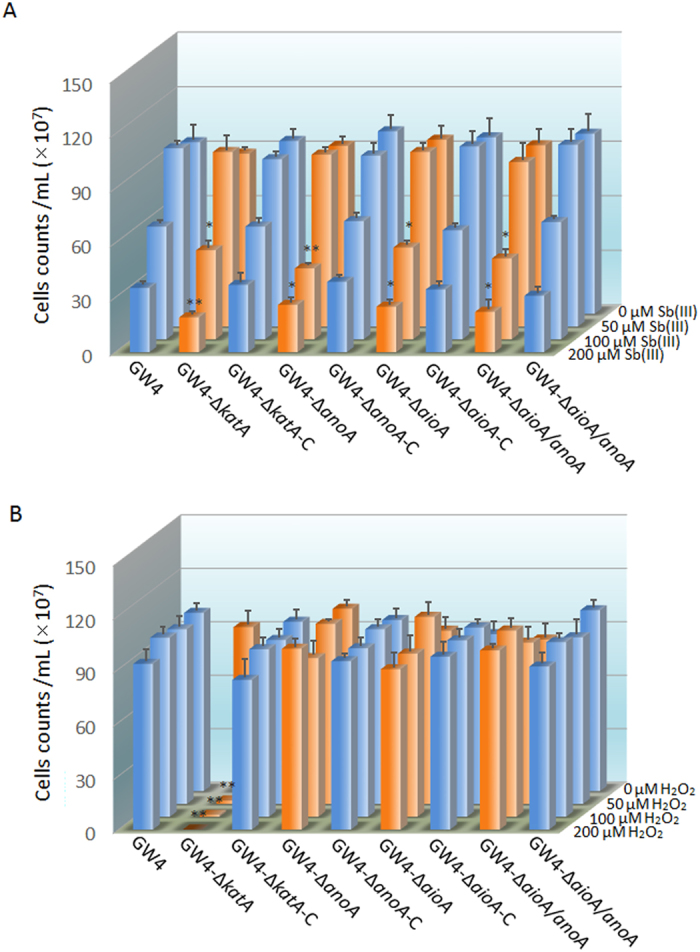
Sb(III) (**A**) and H_2_O_2_ (**B**) resistance of *A. tumefaciens* strains. Bacterial cells were inoculated into 100 mL of CDM medium with the addition of different concentrations of Sb(III) or H_2_O_2_ (0, 50, 100 and 200 μM). After 24 h of incubation, the samples were collected for viable plate counting. Data are shown as the mean of three replicates, with the error bars represents ± SD. **Represents p < 0.01; *represents p < 0.05.

Subsequent efforts focused on the Sb(III) resistance of the *A. tumefaciens* strains. After 48 h incubation in CDM medium without Sb(III) supplementation, all of the strains exhibited a similar amount of viable cell counts (Fig. 4A). Moreover, there was no significant difference between the viable cell counts of the strains cultured with or without 50 μM Sb(III), indicating that 50 μM Sb(III) has no effect on bacterial growth. However, the deletion of *katA* significantly inhibited the growth of strain GW4 with the addition of 100 or 200 μM Sb(III) (p < 0.05 and p < 0.01, respectively), suggesting that the reduced H_2_O_2_ consumption might associated with bacterial Sb(III) resistance. In addition, the growth of strains GW4-Δ*anoA*, GW4-Δ*aioA* and GW4-Δ*aioA*/*anoA* were also obviously constrained (p < 0.05) relative to strain GW4 in the presence of 100 or 200 μM Sb(III), and the phenotypes of the complemented strains were recovered to the wild-type strain (Fig. 4A). These results indicated that both biotic and abiotic factors of Sb(III) oxidation were essential for bacterial Sb(III) resistance.

### The *katA* is involved in H_2_O_2_ resistance

Plate counting assays were also performed to evaluate the antibacterial activities of H_2_O_2_ against *A. tumefaciens* strains using the same culture conditions with Sb(III) resistance. H_2_O_2_ is a substantial component of cellular oxidative stress with a toxic effect on different types of macromolecules^31^,^32^. The growth of strain GW4 was not affected by 50, 100 and 200 μM H_2_O_2_, suggesting that the oxidative stress response in strain GW4 is efficient for the detoxification of such concentrations of H_2_O_2_. For strains GW4-Δ*aioA*, GW4-Δ*anoA* and GW4-Δ*aioA*/*anoA* and their complemented strains, the viable cell counts were consistent with wild-type strain GW4, indicating that the deletion of biotic factors in strain GW4 did not affect bacterial H_2_O_2_ resistance (Fig. 4B). However, the growth of GW4-Δ*katA* was significantly inhibited by 50, 100 and 200 μM H_2_O_2_ (p < 0.01), because such concentrations of H_2_O_2_ could not be efficiently consumed without decomposition through KatA. The phenotype of the complemented strain GW4-Δ*katA*-C was recovered (Fig. 4B). The results demonstrated that *katA* is essential for bacterial H_2_O_2_ resistance.

### Correlation between H_2_O_2_ content and Sb(III) oxidation both *in vivo* and *in vitro*

**Figure 5:**
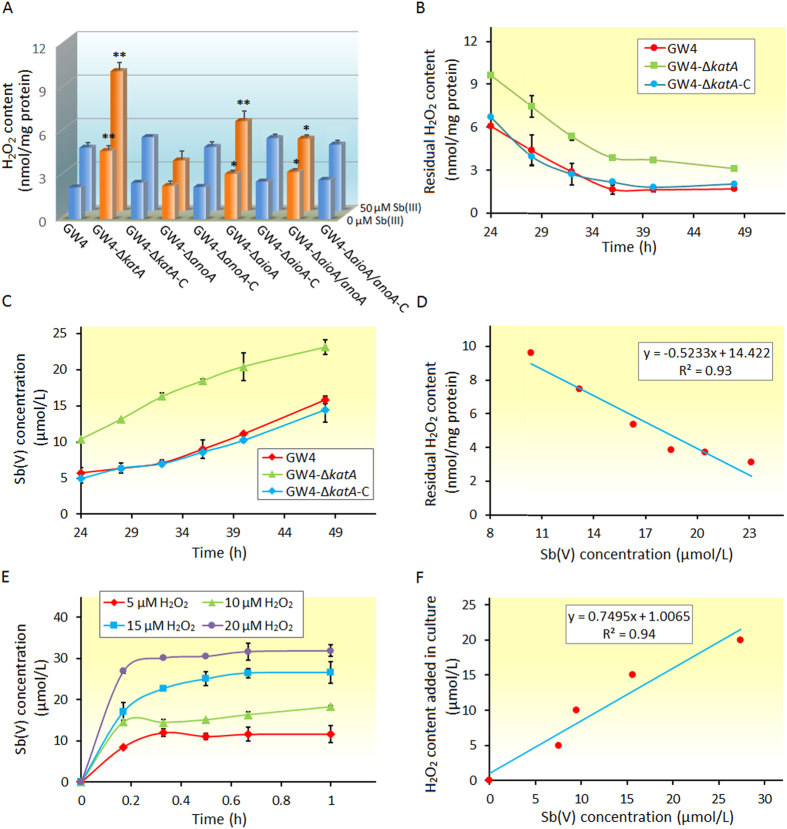
The cellular H_2_O_2_ content is correlated with bacterial Sb(III) oxidation. (**A**) The H_2_O_2_ content and relevant Sb(V) concentration of *A. tumefaciens* strains after 2 h of incubation with or without 50 μM Sb(III). (**B**) H_2_O_2_ content and (**C**) Sb(V) concentration in strains GW4, GW4-Δ*katA* and GW4-Δ*katA*-C from 24 to 48 h of cultivation in CDM medium with the addition of 50 μM Sb(III). (**D**) Correlation between the H_2_O_2_ content and Sb(V) concentration in strain GW4-Δ*katA*. (**E**) Dynamic changes in the H_2_O_2_ and Sb(V) contents in CDM medium with 50 μM Sb(III) and different concentrations of H_2_O_2_ and without the inoculation of *A. tumefaciens* strains. (**F**) Correlation between H_2_O_2_ and Sb(V) contents *in vitro*. Data are shown as the mean of three replicates, with the error bars represents ± SD. **Represents p < 0.01; *represents p < 0.05.

To understand the relationship between the cellular H_2_O_2_ content and Sb(III) oxidation, we examined the H_2_O_2_ content in *A. tumefaciens* strains with or without the induction of 50 μM Sb(III). The results indicated the following: i) The generation of cellular H_2_O_2_ was induced by Sb(III), ii) The cellular H_2_O_2_ content was consistent with the transcription level of genes associated with abiotic Sb(III) oxidation, and iii) The content of H_2_O_2_ was proportional to the bacterial Sb(III) oxidation efficiency. The strain with a higher H_2_O_2_ content showed a faster Sb(III) oxidation efficiency (Figs 2 and 5A). In addition, we measured the dynamic changes in the cellular H_2_O_2_ content and Sb(V) generation in strains GW4, GW4-Δ*katA* and GW4-Δ*katA*-C from 24 to 48 h cultivation with the addition of 50 μM Sb(III). A significant decrease in the residual H_2_O_2_ content was observed with incubation time, while the Sb(V) concentration correspondingly increased (Fig. 5B,C), indicating that the consumed H_2_O_2_ might catalyze bacterial Sb(III) oxidation. Moreover, the H_2_O_2_ content and the increased Sb(V) concentration were significantly linearly correlated, with a correlation coefficient of 0.93 (Fig. 5D).

The dynamic changes in the H_2_O_2_ content and Sb(V) generation were also measured in CDM medium with the addition of 50 μM Sb(III) and different concentrations of H_2_O_2_. Figure 5E showed that Sb(III) was transformed to Sb(V) with the addition of H_2_O_2_, indicating that H_2_O_2_ could oxidize Sb(III) to Sb(V) *in vitro*. In addition, there is a correlation between the H_2_O_2_ and Sb(V) contents *in vitro* (R^2^ = 0.94) (Fig. 5F). Based on the *in vivo* and *in vitro* analyses, we proposed that H_2_O_2_ is responsible for bacterial Sb(III) oxidation as an abiotic oxidant in strain GW4.

### Comparison of Sb(III) oxidation between *A. tumefaciens* GW4 and *A. tumefaciens* 5A

Previous studies have shown that *A. tumefaciens* 5A, which has a *16S rRNA* homology of 99% compared with *A. tumefaciens* GW4, also oxidizes Sb(III) to Sb(V)^17^,^33^. AioAB is responsible for Sb(III) oxidation in strain 5A, as the deletion of *aioA* decreased the Sb(III) oxidation efficiency, in contrast with the phenotype of strain GW4-Δ*aioA*^17^. To clarify the different effects of *aioA* on Sb(III) oxidation between strain GW4 and 5A, we also investigated the *aioA* mutant in strain 5A under the same culture conditions of strain GW4. The growth of strains GW4 and 5A were not affected by disruption of *aioA* in CDM medium supplemented with 50 μM Sb(III) (Fig. S2A,B). However, the Sb(III) oxidation efficiency was increased in strain GW4-Δ*aioA* (Fig. S2C), and the transcription of *anoA* and *katA* and the cellular H_2_O_2_ content were increased when *aioA* was deleted (Figs S3A and S4A). In contrast, the Sb(III) oxidation efficiency was decreased in strain 5A-Δ*aioA* (Fig. S2D), and no increased transcription of *katA* was observed, moreover, the transcription level of *anoA* was only slightly increased (Fig. S3B). Although Sb(III) also stimulated the generation of H_2_O_2_, this process was not affected by the disruption of *aioA* in strain 5A (Fig. S4B).

## Discussion

Currently, studies have shown that bacterial Sb(III) oxidation is catalyzed through AioAB or AnoA with a certain percentage of contribution^17^,^18^, indicating the existence of other new bacterial Sb(III) oxidation mechanisms. The present study documents a non-enzymatic basis for microbial Sb(III) oxidation, according to the following observations: i) The transcription of *katA, sod1, sod2* and the cellular H_2_O_2_ content were induced by Sb(III); ii) the Sb(III) oxidation efficiency was consistent with the cellular H_2_O_2_ content in *A. tumefaciens* strains; and iii) The cellular H_2_O_2_ content in the *katA* mutant was remarkably linearly correlated with the Sb(V) concentration. Thus, we concluded that the cellular H_2_O_2_ acts as an abiotic factor in bacterial Sb(III) oxidation. The cellular H_2_O_2_ mediated bacterial Sb(III) oxidation is an smart detoxification process of Sb(III)-oxidizing bacteria through the “using poison against poison” strategy, which could transform the toxic Sb(III) to the much less toxic Sb(V) and consume the toxic cellular H_2_O_2_ simultaneously.

In addition, the Sb(III) resistance mechanism associated with bacterial oxidative stress has not been well clarified so far. It has been reported that H_2_O_2_ induces the death of *E. coli*, primarily reflecting DNA damage via the Fenton reaction^34^,^35^,^36^. Recently, the bacterial oxidative stress was found to associate with Sb(III) oxidation in *Pseudomonas stutzeri* TS44^37^. In this study, the deletion of *katA* significantly decreased H_2_O_2_ resistance, reflecting the disruption of the release of the oxidative stress response in strain GW4. A large number of literatures have shown that heavy metals (e.g. Cr, Cd)^38^,^39^, transition metals (e.g. Fe, Cu)^23^ and metalloid (As)^40^ could induce bacterial oxidative stress response due to their toxic effects. As a most common toxic heavy metal, the production of H_2_O_2_ could be the primary response of bacterial to Sb(III). It appears that the *katA* mutant is more tolerant to Sb(III) than H_2_O_2_ because Sb(III) oxidation consumed the cellular H_2_O_2_ even the *katA* was disrupted. In the presence of 50 μM Sb(III), the amount of H_2_O_2_ was consumed by Sb(III) oxidation and the cellular H_2_O_2_ was not toxic enough to inhibit bacterial growth. However, in the presence of high concentrations (e.g. 100 or 200 μM) of Sb(III), the high amount of H_2_O_2_ induced by Sb(III) in the *katA* mutant has a higher toxic effect on bacterial cells, even though the Sb(III) oxidation also consumed some of the H_2_O_2_. Thus, the Sb(III) resistant level in strain GW4-Δ*katA* was still lower than the wild-type strain GW4.

To understand bacterial Sb(III) oxidation and the contribution of each cellular oxidative factor comprehensively, we also investigated the effects of biotic factors on Sb(III) oxidation in *A. tumefaciens* GW4. The deletion of *anoA* led to a ~ 30% decrease in the Sb(III) oxidation efficiency and the abiotic Sb(III) oxidation was not enhanced, indicating that the decreased Sb(III) oxidation efficiency reflected the contribution of AnoA. However, the deletion of *aioA* increased Sb(III) oxidation efficiency, reflecting the increased expression of AnoA and the generation of more H_2_O_2_. Thus, the contribution of AioAB to Sb(III) oxidation in strain GW4 was not obvious, however, AioAB is indeed related to Sb(III) oxidation, since it affects the expressions of AnoA and KatA. In addition, the results of a kinetic analysis in our previous study indicated that AnoA tends to catalyze the Sb(III) oxidation more efficiently than As(III) oxidation, while AioAB is prone to catalyze As(III) oxidation^16^. These results suggested that the effect of AnoA on Sb(III) oxidation may higher than that of AioAB. The contribution of H_2_O_2_-catalyzed abiotic Sb(III) oxidation may higher than that of enzymatic catalysis in strain GW4 since the disruption of KatA significantly increased Sb(III) oxidation efficiency^16^.

The results of a previous study demonstrated that AioAB was responsible for bacterial Sb(III) oxidation in *A. tumefaciens* 5A, suggesting that the effect of *aioA* on Sb(III) oxidation between strain 5A and GW4 was different. In strain 5A, AioAB has a substantial contribution to Sb(III) oxidation efficiency (approximately 25%)^17^. However, it appears that AioAB has no positive effect on Sb(III) oxidation in strain GW4, potentially reflecting the different characteristics between these two strains. The Sb(III) MIC of strain 5A is 0.3 mM, while strain GW4 is a highly Sb(III) resistance bacterium with a 8 mM MIC of Sb(III) (data not shown). In addition, strain 5A had a longer lag phase than that of strain GW4 with the addition of 50 μM Sb(III), indicating that Sb(III) might has a more toxic effect on strain 5A. Nonetheless, we cannot exclude the possibility that AioA might catalyze Sb(III) oxidation along with AnoA in strain GW4 because complex regulatory mechanism(s) might be involved in the compensation of the Sb(III) oxidation efficiency in the *aioA* mutant, which needs to be further studied.

In nature, H_2_O_2_ is a strong oxidant and it can oxidize not only Sb(III), but also other metalloids, such as As(III). However, the efficiency of bacterial As(III) oxidation catalyzed by H_2_O_2_ is not as obvious as Sb(III) oxidation and the abiotic As(III) oxidation could be hardly observed *in vivo* (data not shown). So far, bacterial As(III) oxidation has been found to be an enzymatic reaction which is primarily catalyzed through AioAB in most As(III)-oxidizing bacteria^41^. However, the mechanism of bacterial Sb(III) oxidation is a co-metabolism process catalyzed by AioAB, AnoA and H_2_O_2_, which is different from bacterial As(III) oxidation (at least in *A. tumefaciens* GW4). Although previous studies have shown that AnoA could also oxidize As(III) *in vitro*^16^, the expression of *anoA* was not induced by As(III)^18^, and the deletion of *anoA* did not affect the As(III) oxidation efficiency in strain GW4 (data not shown). Thus, the effect of AnoA on bacterial As(III) oxidation was hardly observed. In addition, H_2_O_2_ is an important and effective oxidant responsible for Sb(III) oxidation in alkaline aqueous environments^42^,^43^, and the Sb(III) oxidation rate is much faster than that of As(III)^44^,^45^,^46^. In the present study, the pH of the cultures increased from the initial 6.5 to approximately 8.0 following exposure to Sb(III), indicating that the culture conditions are suitable for H_2_O_2_ to catalyze Sb(III) oxidation (Fig. S3E,F). However, the culture pH decreased with the increasing incubation time during As(III) oxidation of strain GW4 (data not shown), and this pH might not be suitable for abiotic oxidation mediated through H_2_O_2_. Therefore, the abiotic oxidation is more effective on Sb(III) in strain GW4.

**Figure 6:**
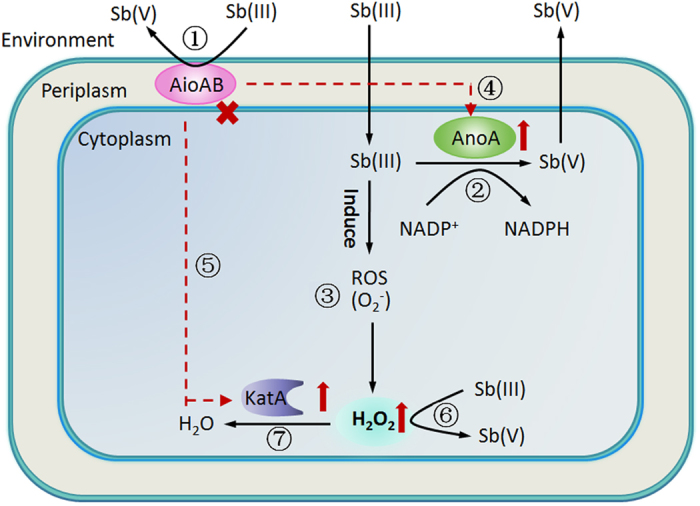
A proposed model of bacterial Sb(III) oxidation based on the present study and literatures. (1) Sb(III) oxidation might be catalyzed through As(III) oxidase AioAB^17^; (2) Sb(III) oxidase AnoA was shown to oxidize Sb(III) to Sb(V) in cytoplasm with NADP^+^ as a cofactor^18^; (3) Sb(III) induced the cellular production of ROS (O_2_^−^) and H_2_O_2_; (4–5) Deletion of *aioA* in *A. tumefaciens* GW4 resulted in the increased expression of AnoA and KatA and cellular H_2_O_2_ content; (6) H_2_O_2_ oxidizes Sb(III) oxidation as an abiotic factor; (7) The residual H_2_O_2_ was partially consumed through KatA.

Based on the observations of the present study, we proposed that microbial Sb(III) oxidation is a co-metabolism process in strain GW4 (Fig. 6): (i) AioAB might be responsible for Sb(III) oxidation in the periplasm^17^; (ii) AnoA catalyzes cytoplasmic Sb(III) oxidation with NADP^+^ as a co-factor^18^; (iii) Sb(III) induces the bacterial oxidative stress response, leading to the production of ROS^37^ and H_2_O_2_; iv) the disruption of AioAB increases the expression of AnoA; (v) the disruption of AioAB increases the cellular H_2_O_2_ content and expression of KatA; (vi) the induced H_2_O_2_ oxidizes Sb(III) to Sb(V); and (vii) the redundant H_2_O_2_ is partially consumed by KatA.

In summary, the present study provides novel evidences that microbial antimonite oxidation contains both abiotic and biotic mechanisms and elucidates the contribution of each oxidative factor. We show that Sb(III) causes oxidative stress to bacterial cells and further induces the generation of cellular H_2_O_2_. Sb(III) oxidation is a detoxification process by transforming the toxic Sb(III) to the much less toxic Sb(V). Meanwhile, since the cellular H_2_O_2_ is consumed by Sb(III) oxidation process, the Sb(III) oxidation also contributes to against the toxic H_2_O_2_. Such co-mechanism may be widely exist in other Sb(III)-oxidizing microorganisms. The relationship among the biotic and abiotic factors may be further studied by changing Sb(III) concentration, environmental and nutritious conditions.

## Materials and Methods

### Strains and genomic analysis

Bacterial strains and plasmids used in the present study are listed in Table S1. *A. tumefaciens* strains were grown in a chemically defined medium (CDM)^47^ containing 0 or 50 μM K_2_Sb_2_(C_4_H_2_O_6_)_2_ [Sb(III)] with aeration through shaking at 28 °C. *E. coli* strains were cultured at 37 °C in Luria-Bertani (LB) medium. When required, ampicillin (Amp, 100 mg/mL), kanamycin (Kan, 50 mg/mL), tetracycline (Tet, 5 mg/mL), gentamicin (Gen, 50 mg/mL) or chloromycetin (Cm, 50 mg/mL) were added. The genomic analyses of *aioA, anoA, katA* and *sod* were conducted through blastn and blastp in the genome of *A. tumefaciens* GW4 on the NCBI website (http://www.ncbi.nlm.nih.gov).

### Constructions *A. tumefaciens* GW4 mutant strains and complemented strains

An in-frame deletion in *katA* was constructed in strain GW4 using crossover PCR^48^. The primers used for construction of the deletion are listed in Table S2. The PCR products were double digested with *BamH*I and *Xba*I and subsequently cloned into pJQ200SK digested with the same restriction enzymes. The final construct pJQ-*katA* was mobilized into strain GW4 via conjugation with *E. coli* S17-1. Single-crossover mutants were identified on LB agar plates containing 100 μg/mL Amp and 50 μg/mL Gen, which were subsequently screened on CDM agar containing 20% sucrose^49^. Sucrose^R^ and Gen^Sen^ transconjugants were screened using PCR and DNA sequencing to verify the *katA* deletion. For GW4-Δ*aioA*/*anoA*, an in-frame deletion in *anoA* was constructed in the mutant strain GW4-Δ*aioA* using the method described above. The GW4-Δ*aioA* and GW4-Δ*anoA* mutants and their complementary strains were obtained from previous works^17^,^18^.

The construction of GW4-Δ*katA* complementation was accomplished using plasmid pCPP30. The complete *katA* coding region was PCR-cloned into *BamH*I - *Pst*I double-digested pCPP30. The resulting plasmid pCPP30-*katA* was subsequently mobilized into strain GW4-Δ*katA* via *E. coli* S17-1. Tet^R^ and Amp^R^ transconjugants were screened on LB agar plates, yielding the complementary strain GW4-Δ*katA*-C. The complementation of GW4-Δ*aioA*/*anoA* was performed using two plasmids, pCPP30 and pCT-Zori^50^. The *aioAB* genes along with the upstream RpoN binding site and the complete *anoA* coding region were PCR-cloned into *BamH*I - *Pst*I double-digested pCT-Zori and pCPP30, respectively. The resulting plasmid pCT-Zori-*aioAB* and pCPP30-*anoA* were simultaneously mobilized into strain GW4-Δ*aioA*/*anoA* via *E. coli* S17-1. Subsequently, Tet^R^, Cm^R^ and Amp^R^ transconjugants were screened on LB agar plates. The complementary strains were verified through PCR and DNA sequencing.

### Quantitative RT-PCR analysis

To investigate the expression of the genes associated with Sb(III) oxidation in *A. tumefaciens* strains, overnight cultures of these strains were each inoculated into 100 mL of CDM at 28 °C with 120 rpm shaking. When the OD_600_ reached 0.2–0.3, 0 or 50 μM Sb(III) was added to the cultures. After 0.5 h of induction, the bacterial cells were harvested for total RNA extraction using Trizol reagent (Invitrogen) and treated with RNase-free DNase I (Takara) according to the manufacturer’s instructions (Invitrogen, Grand Island, NY, USA). The quality and quantity of the RNA were monitored using a spectrophotometer (NanoDrop 2000, Thermo). Reverse transcription was performed using the RevertAid First Strand cDNA Synthesis Kit (Thermo) with 300 ng total RNA for each sample^51^. Subsequently, the obtained cDNA was diluted 10-fold and used as a template for further analysis. Quantitative RT-PCR was carried out by ABI VIIA7 in 0.1 mL Fast Optical 96-well Reaction Plate (ABI) using SYBR^®^ Green Real-time PCR Master Mix (Toyobo) and the primers listed in Table S2. To eliminate error, three technical and biological replicates were established for each reaction. The *A. tumefaciens* GW4 16S rRNA gene was used as an internal control and the expression data of the genes were normalized to 16S rRNA without Sb(III) using the formula 2^−(ΔCT−CT,16SrRNA,zero Sb)^, which was modified from the 2^−ΔΔCT^ method^52^,^53^,^54^.

### Growth, Sb(III) oxidation and sensitivity assays

*A. tumefaciens* strains were each inoculated into 5 mL of CDM with the addition of 50 μM Sb(III) and incubated at 28 °C with shaking at 120 rpm. When the OD_600_ reached 0.5–0.6, the strains were each inoculated into 100 mL of CDM in the presence of 50 μM Sb(III). Culture samples were collected every 8 h for measuring OD_600_ by spectrophotometry (DU800, Beckman). In addition, the samples were centrifuged (13,400 × g) and subsequently filtered (0.22 μm filter) to monitor the Sb(III)/Sb(V) concentrations through HPLC-HG-AFS (Beijing Titan Instruments Co., Ltd., China) according to Li *et al*.^55^.

To determine the Sb(III) and H_2_O_2_ resistance of *A. tumefaciens* strains, the viable plate counting method was employed. The strains were each inoculated into 100 mL of CDM medium with the addition of different concentrations of Sb(III) or H_2_O_2_ (0 μM, 50 μM, 100 μM and 200 μM) respectively, and incubated at 28 °C with shaking at 120 rpm. After cultivation for 48 h, the samples were collected for gradient dilution and spread onto solid LB medium, respectively. The plates were incubated at 28 °C and counted after 2–3 days until colonies formed.

### H_2_O_2_ content and Sb(III) oxidation assays

To assess the H_2_O_2_ contents, *A. tumefaciens* strains were cultured as described above. After incubation for 0.5 h with 50 μM Sb(III), the bacterial cells (2 mL) were harvested through centrifugation (13,400 × g for 5 min at 4 °C) and washed twice with 50 mmol/L K_3_PO_4_ (pH 7.8). Subsequently, the cells were resuspended in 1 mL K_3_PO_4_ (pH 7.8) and sonicated on ice. The supernatants were obtained through centrifugation (13 400 × g, 10 min, 4 °C) to remove cell debris and subsequently mixed with 50 μL amplex red (AR) (Chemical Co., St. Louis, MO, USA) and 50 μL horseradish peroxidase (HRP) (F. Hoffmann-La Roche Ltd, Shanghai, China)^56^. After incubation at 37 °C for 15 min, fluorescence (530 ex/587 em) was measured using an EnVision^®^ Multimode Plate Reader (Perkin Elmer).

To determine the dynamic variations in the H_2_O_2_ and Sb(V) contents *in vivo*, strains GW4, GW4-Δ*katA* and GW4-Δ*katA*-C were each inoculated into 100 mL of CDM medium supplemented with 50 μM Sb(III) and incubated at 28 °C for 48 h with shaking at 120 rpm. At designated times, the culture samples were collected to assess the H_2_O_2_ contents and monitor the Sb(V) contents as described above. For *in vitro* dynamic changes in the H_2_O_2_ and Sb(V) contents, at designated times, the measurement of Sb(V) concentrations was performed in CDM medium with the addition of 50 μM Sb(III) and different concentrations of H_2_O_2_ (5 μM, 10 μM, 15 μM and 20 μM).

## Additional Information

**How to cite this article**: Li, J. *et al*. Abiotic and biotic factors responsible for antimonite oxidation in *Agrobacterium tumefaciens* GW4. *Sci. Rep.* **7**, 43225; doi: 10.1038/srep43225 (2017).

**Publisher's note:** Springer Nature remains neutral with regard to jurisdictional claims in published maps and institutional affiliations.

## Supplementary Material

Supplementary Information
