# Supplementary material for: Abiotic and biotic factors responsible for antimonite oxidation in Agrobacterium tumefaciens GW4
Source: Sci Rep. 2017 Mar 2;7:43225. doi: 10.1038/srep43225 (PMC5333119; doi:10.1038/srep43225)
Supplement: Supplementary Information [file srep43225-s1.pdf]

Abiotic and biotic factors responsible for antimonite oxidation in *Agrobacterium tumefaciens* GW4

Jingxin Li, Birong Yang, Manman Shi, Kai Yuan, Wei Guo, Qian Wang, Gejiao Wang*

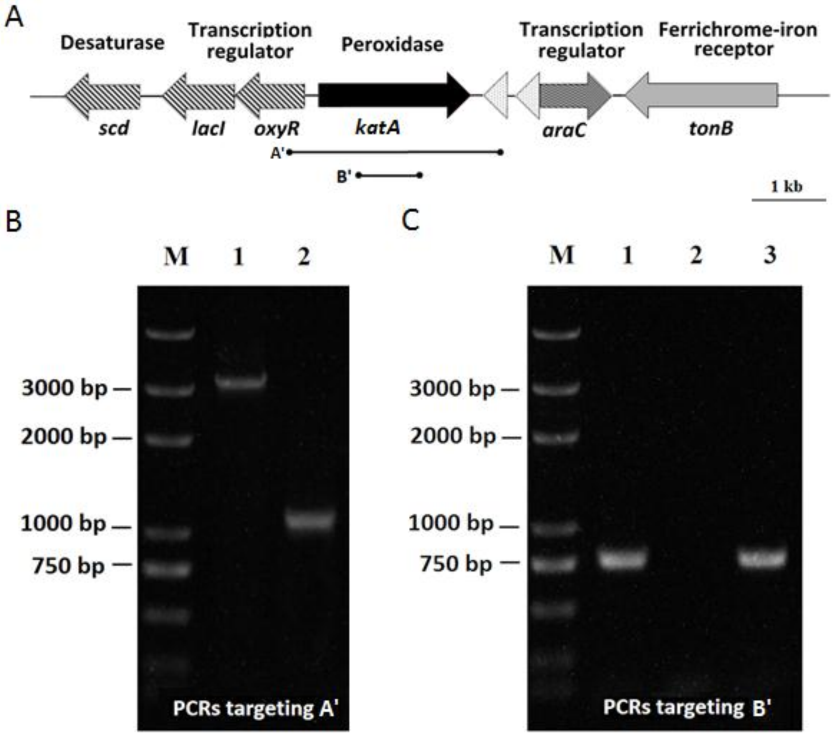

Figure S1. Physical map (A) and evidence of *katA* deletion and its complementation (B-C). (A) Gene cluster of *katA*. A′ designates a 3,170 bp DNA fragment targeted in diagnostic PCR with primers PMkatA-1F/PMkatA-1R (Supporting Information Table S2) and depicted in panel B; B′ designates a 770 bp DNA fragment targeted in diagnostic PCR with primers PMkatA-2F/PMkatA-2R (Supporting Information Table S2) and depicted in panel C. (B-C) Diagnostic PCR confirming the deletion of *katA* to create mutant strain GW4-Δ*katA* and complementation to create GW4-Δ*katA*-C. For panels B and C: Lane 1,

WT strain; lane 2, mutant strain GW4-Δ*katA*, and lane 3, the complementary strain GW4-Δ*katA*-C. M, the molecular weight marker (DL 2000 plus). Amplicon identities were confirmed by DNA sequencing.

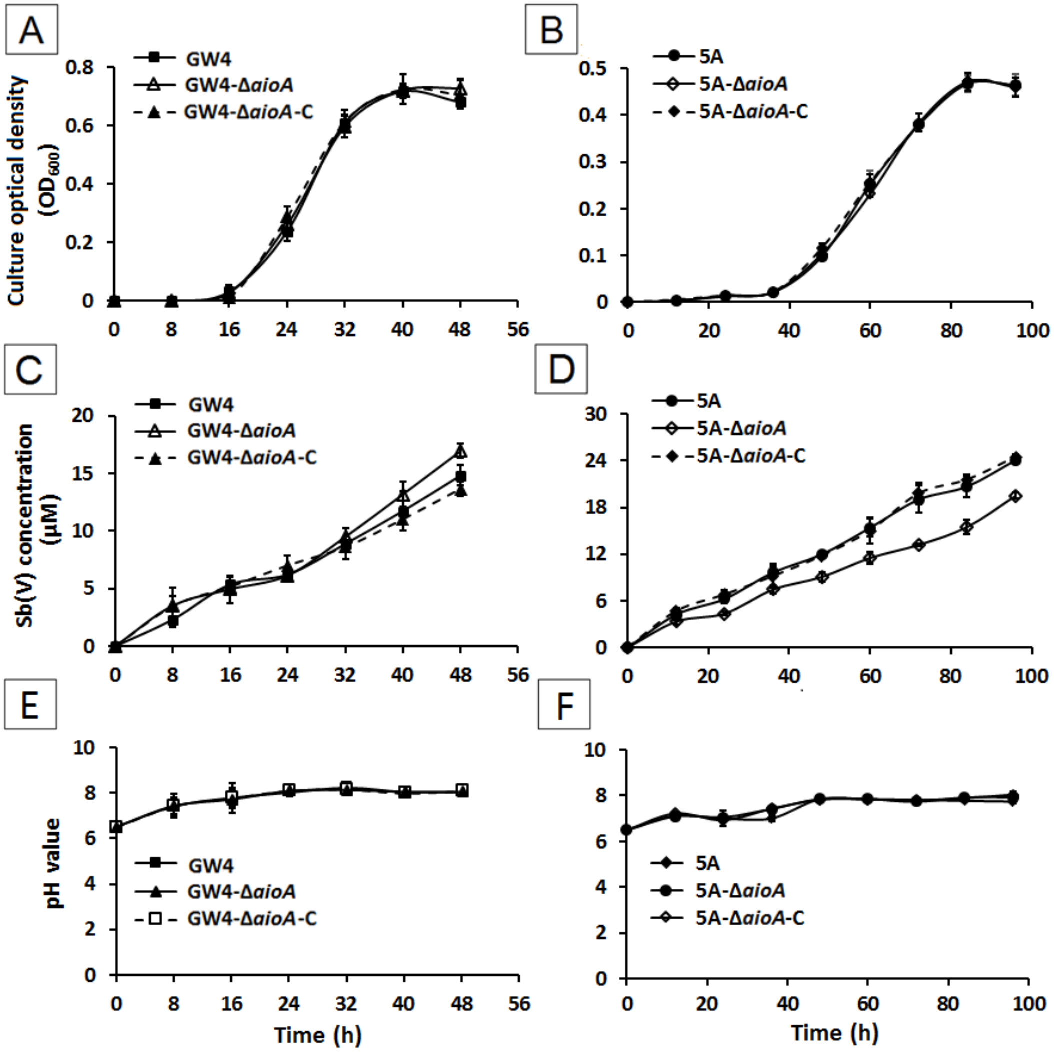

Figure S2. Influences of *aioA* genotype on the growth and Sb(III) oxidation of *A. tumefaciens* GW4 and 5A. (A, B) The growth of strain GW4 and 5A were not affected by the *aioA* genotype with the addition of 50 μM Sb(III) in CDM medium. (C, D) Different effects of *aioA* genotype on Sb(III) oxidation between strain GW4 and 5A. Cell growth was measured based on the culture optical density, and Sb(V) concentrations in the culture

fluids were measured using HPLC-HG-AFS. (E, F) The pH value of strain GW4 and 5A during the growth in CDM medium with the addition of 50 μM Sb(III). Error bars correspond to the standard deviations of the means from three independent experiments.

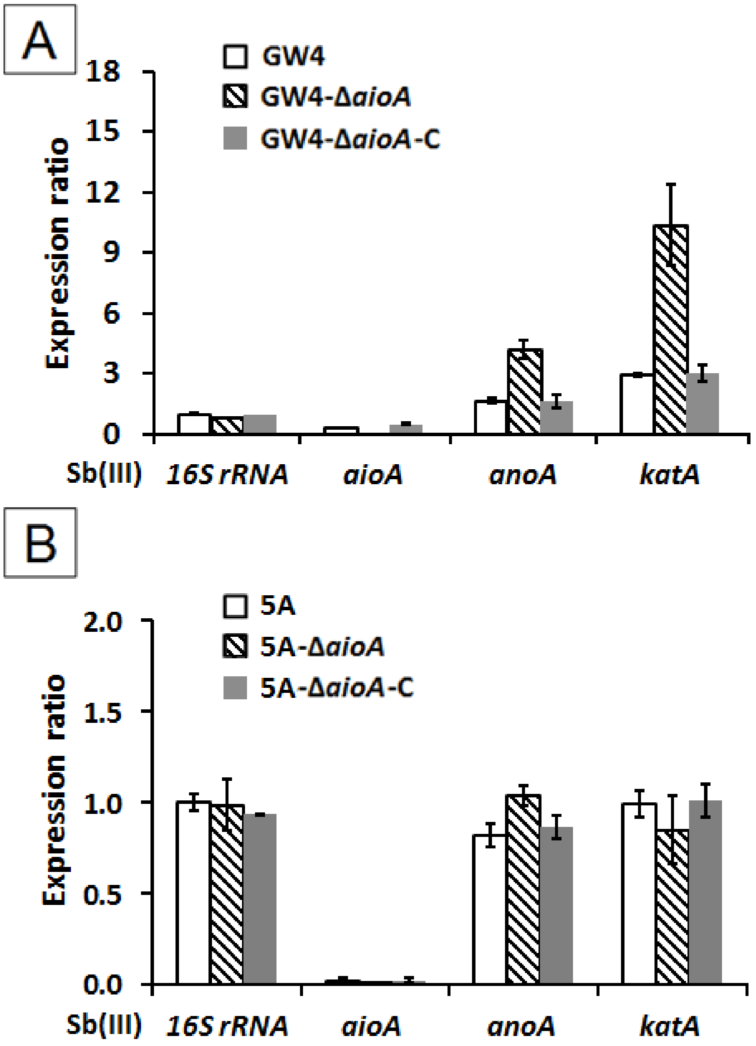

Figure S3. Quantitative reverse transcriptase-PCR analysis of the genes associated with Sb(III) oxidation in *A. tumefaciens* GW4 (A) and 5A (B). Total RNA was each isolated from *A. tumefaciens* strains after 2 h of incubation with 50 μM Sb(III). The 16S rRNA gene was used as a reference. Data are shown as the mean of three replicates, with the error bars representing ± SD.

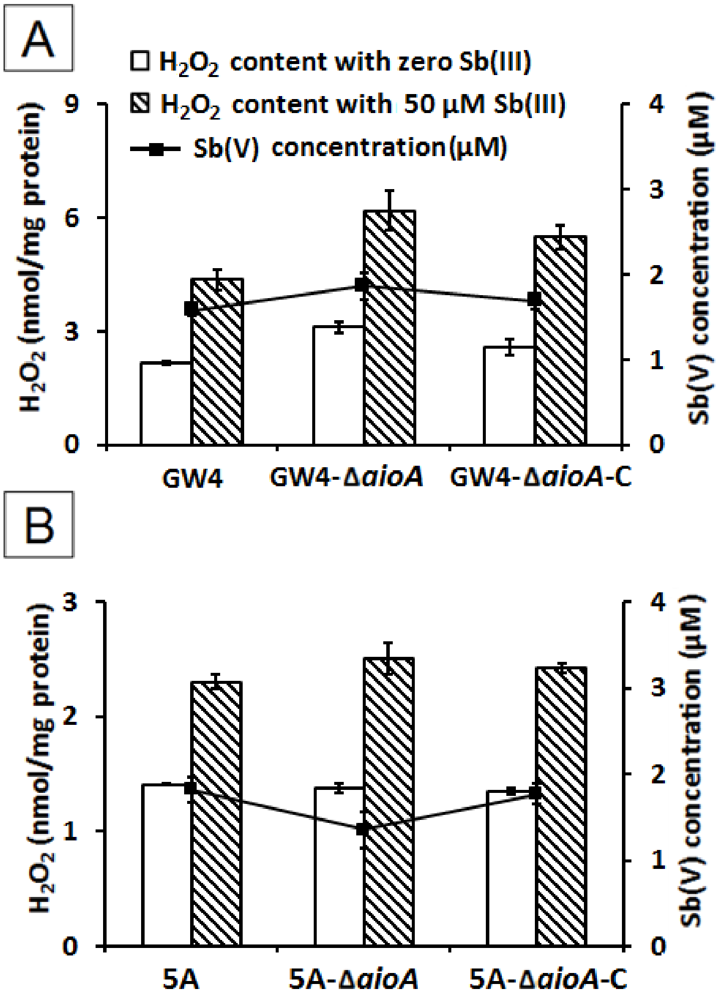

Figure S4. Quantitative reverse transcriptase-PCR analysis of the genes associated with Sb(III) oxidation in *A. tumefaciens* GW4 (A) and 5A (B). Total RNA was each isolated from *A. tumefaciens* strains after 2 h of incubation with 50 μM Sb(III). The 16S rRNA gene was used as a reference. Data are shown as the mean of three replicates, with the error bars representing ± SD.

Table S1. The strains and plasmids used in the present study

Strain/plasmid	Relevant properties or derivation	Source or reference
**Strains**		
Agrobacterium tumefaciens		
GW4	Wild type, As(III) oxidizing, Sb(III) oxidizing	Fan *et al*., 2008
GW4-Δ*katA*	*katA* gene deleted	This study
GW4-Δ*katA*-C	Δ*katA* (pCPP30::*katA*) Complementation of Δ*katA*	This study
GW4-Δ*anoA*	*anoA* gene deleted	Li *et al*., 2015
GW4-Δ*anoA*-C	Δ*anoA* (pCPP30::*anoA*) Complementation of Δ*anoA*	Li *et al*., 2015
GW4-Δ*aioA*	*aioA* gene deleted	Wang *et al*., 2015
GW4-Δ*aioA*-C	Δ*aioA* (pCPP30::*aioA*) Complementation of Δ*aioA*	Wang *et al*., 2015
GW4-Δ*aioA*/*anoA*	*aioA* and *anoA* gene deleted	This study
GW4-Δ*aioA*/*anoA*-C	Δ*aioA/anoA* (pCPP30::*anoA*, pCT-Zori::*aioA*) Complementation of Δ*aioA/anoA*	This study
Escherichia coli		
DH5α	*supE*44 *lacU*169(*φ80lacZM15*) *hRDR*17 *recA*1 *endA*1 *gyrA96 thi-1 relA*1	Hanahan, 1983
S17-1 λ*pir*	F^- RP4-2-Tc::Mu *aphA*::Tn7 *recA* λ*pir* lysogen; Sm^R Tp^R	Simon *et al*., 1983
**Plasmids**		
pGEM-T	TA cloning vector; Amp^R	Promega
pJQ200SK	*sacB sacR* Suc^S; Gen^R	Quandt and Hynes, 1993
pJQ(*katA*)	Gene mutation plasmid to create Δ*katA*; *sacB sacR* Suc^S; Gen^R	This study
pJQ(*anoA*)	Gene mutation plasmid to create Δ*anoA*; *sacB sacR* Suc^S; Gen^R	Li *et al*., 2015
pCPP30	Broad host range; Tet^R	Liu *et al*., 2012
pCPP30(*katA*)	Complemented plasmid for Δ*katA*; Tet^R	This study
pCPP30(*anoA*)	Complemented plasmid for Δ*aioA*/*anoA*; Tet^R	Li *et al*., 2015
pCT-Zori(*aioBA*)	Complemented plasmid for Δ*aioA*/*anoA*; Cm^R	This study

Table S2. Primers used in the present study

Primer pair	Primer sequence	Use
PkatA-1F / PkatA-1R	5'AAATCTAGAGCGACGGTGGGAATGACA3'/ 5'**GCGCATAAACTTCCGCATAGG**CGCCCTCCGATGGGTTTA3'	Crossover PCR to create deletion in *katA*
PkatA-2F / PkatA-2R	5'**CCTATGCGGAAGTTTATGCGC**AGGACGACAGCAGGGAGAA3'/ 5'AAAGGATCCGGGTGAGCGTATGGAAAGA3'	Crossover PCR to create deletion in *katA*
CkatA-F / CkatA-R	5'AAACTGCAGATCGGAGGGCGAAATGGAC 3'/ 5'AAATCTAGACCTTGCCAACCCAGATAGAA3'	Complementation for *katA* mutant
PMkatA-1F / PMkatA-1R	5'GCGACGGTGGGAATGACA3'/ 5'GGGTGAGCGTATGGAAAGA 3'	Confirmation of *katA* disruption and complementary
PMkatA-2F / PMkatA-2R	5'AGCACGACACCAAGACCAATC3'/ 5'TTTCAGGGTCTCGGCTGG3'	
16S-F 16S-R	5' GGTATGGGCATTGGAGACGA3'/ 5' GGTATGGGCATTGGAGACGA3'	Real-time RT-PCR
katA-F katA-R	5'CGGCCTCGGGTAAATCG3'/ 5'CGGCCTTCAATGCTTCGAT3'	Real-time RT-PCR
anoA-F anoA-R	5'TCATGGTCGAAAGCATCGG3'/ 5'GCGGTTCTGGACGATGTCATA3'	Real-time RT-PCR
aioA-F aioA-R	5'AGCACAATGTCACCTGCCACTT3'/ 5'TCCGCCTGCTGTTGTTCC3'	Real-time RT-PCR

* The bolded sequences denote the reverse complement sequences for the crossover PCR, while the underlined sequence denotes the restriction enzyme sites.
